# Occurrence and vertical transmission of avian polyomavirus and circovirus in captive and wild Passeriformes in Poland

**DOI:** 10.1186/s12917-025-04899-2

**Published:** 2025-07-07

**Authors:** Aleksandra Ledwoń, Ines Szotowska, Izabella Dolka, Joanna Turniak

**Affiliations:** Department of Pathology and Veterinary Diagnostics, Institute of Veterinary Medicine, Warsaw University of Life Sciences (WULS-SGGW), Nowoursynowska 159c, Warsaw, 02-776 Poland

**Keywords:** Passeriformes, Passerine birds, Circovirus, Avian polyomavirus, Co-infection, Vertical transmission

## Abstract

**Background:**

Diseases caused by polyomaviruses and circoviruses in parrots were first described in the 1980s. Then they began to be diagnosed in other orders of birds, including Passeriformes, such as Atlantic canaries (*Serinus canaria*) and estrildid and fringillid finches. Over time, these viruses have also been found in an increasing number of captive and wild passerine species. The aim of the study was to assess the occurrence of these viruses in captive and wild Passeriformes in Poland, and the transmission of polyomaviruses and circoviruses through eggs in Atlantic canaries and Bengalese munias (*Lonchura striata domestica*).

**Results:**

Nested PCR tests for avian polyomavirus (APyV) and circovirus (CV) were carried out on organ samples from 331 captive and wild birds belonging to 45 species, necropsied between 2006 and 2024. Additionally, 112 samples of eggs and dead chicks of Atlantic canaries and Bengalese munias from two aviaries with breeding problems were examined. Positive PCR results for APyV were found in 98 birds (29.6%), while CV DNA was detected in 152 birds (45.9%). In 104 wild birds examined, APyV was found in 13 (12.5%) and CV in 35 (33.7%) individuals. In 227 captive birds, APyV was found in 85 (37.4%) and CV in 117 (51.5%) individuals. Co-infections with both viruses were found in 25.1% of captive birds and 7.7% of wild birds. Negative results for both APyV and CV were found in 145 (43.8%) birds tested. In Atlantic canaries, CV DNA was identified in 79% of unfertilized eggs and 59% of embryos examined. In Bengalese munias, CV DNA was identified in 62.5% of unfertilized eggs and in all chicks examined. APyV DNA was not detected in eggs or embryos of canaries and Bengalese munias, nor in any Bengalese munia chicks or canary chicks younger than 7 days.

**Conclusions:**

Avian polyomaviruses and circoviruses are widespread in the population of captive and wild passerines in Poland, and a higher percentage of birds are infected with circovirus than with polyomavirus. Co-infections are more commonly observed in captive passerine birds than in wild passerine birds. Vertical transmission occurs for circoviruses, but not for polyomaviruses, in Atlantic canaries and Bengalese munias.

## Background

The first cases of avian polyomavirus (APyV)-related disease in passerines were diagnosed in the 1980s in captive Gouldian finches (*Erythrura gouldiae*) in Australia [1]. Further cases were reported in the 1990s, primarily in Europe and the USA, affecting captive Gouldian finches, painted finches (*Emblema pictum*), Atlantic canaries (*Serinus canaria*), goldfinches (*Carduelis carduelis*), green finches (*Chloris chloris*), white-rumped shama (*Copsychus malabaricus*), seedcrackers (*Pyrenestes* spp.), and blue bills (*Spermophaga haematina*) [2–7]. Research conducted in Germany from 2000 to 2004 revealed APyV infections in passerine birds, including collared grosbeaks (*Mycerobas affinis*), Eurasian bullfinches (*Pyrrhula pyrrhula griseiventris*), brown bullfinches (*Pyrrhula nipalensis*), grey-headed bullfinches (*Pyrrhula erythaca*), and yellow-bellied tits (*Periparus venustulus*) [8]. Additionally, Rinder et al. [9] demonstrated APyV in red avadavat (*Amandava amandava*), red-browed finch (*Neochmia temporalis*), red-cheeked cordonbleu (*Uraeginthus bengalus*), star finch (*Neochmia ruficauda*), white-rumped seedeater (*Serinus leucopygius*) and zebra finch (*Taeniopygia guttata*) [9]. In Poland, APyV infection in Passeriformes was first described in Atlantic canaries in 2015 [10]. In 2016, a case of polyomavirosis in a flock of white-headed munias (*Lonchura maja*) in a Hungarian aviculturist was reported [11]. In wild birds, APyV was first described in corvids in 2006, in cases of increased mortality due to enteritis in jackdaws (*Corvus monedula*) in Spain. In addition to APyV, *Salmonella* spp. were also detected in these birds [12]. In 2014, polyomavirus was described in Australia in a wild grey butcherbird (*Cracticus torquatus*) [13]. Further cases of APyV infection in jackdaws were recorded in Poland in 2016 when a positive result was obtained in 4 out of 10 tested birds [14].

Clinical signs of polyomavirosis in captive passerine birds are primarily observed during the breeding season [7]. In fledgling chicks and young adults, nonspecific disease symptoms occur 24 to 48 h before death [1] or may show sudden death [5, 7]. Birds that survived the initial acute phase of the disease may develop a chronic form of polyomavirosis, characterized by poor development of the young birds, abnormal feather growth, and beak deformations (tubular shaped mandibles) [5, 7]. In passerines in Germany, finch polyomavirus (FPyV) infection has been associated with dermatitis starting on the base of the beak and spreading over the head, observed a few days before death [8]. Periocular skin lesions have been diagnosed in grey butcherbird (*Cracticus torquatus*) [13]. The most characteristic necropsy changes are hepatomegaly, splenomegaly, and nephromegaly [1, 5, 10, 11]. Typical histopathological findings were observed in the liver, kidneys, lungs, and intestines [11]. Infected cells may exhibit basophilic to amphophilic intranuclear inclusion bodies suggestive of polyomavirus and are often necrotic and swollen [1, 7, 10]. In 2023, a case of T-cell lymphoma in a colony of zebra finches associated with polyomavirus infection was described [15], this opens a new chapter on the pathogenicity of polyomaviruses as oncogenic to birds, for which they were known in human medicine [16]. The transmission of polyomaviruses via eggs is still debated, although it does not appear to occur in parrots [17]. The available literature does not contain studies on this subject in passerines, however, the vertical transmission has also been studied in Anseriformes, but the results are also not conclusive [18]. Diagnosis of avian polyomavirus was originally based on the detection of characteristic intranuclear inclusion bodies by light microscopy or typical virus particles by electron microscopy. Serological diagnostics or APyV-derived nucleotide probes were also used in immunohistochemical and in situ hybridization experiments, respectively [9]. Currently, detection of APyV infection is most often based on PCR tests, which, together with clinical changes, gross lesions and microscopy, lead to the diagnosis of the disease [17].

Circovirus (CV) infection in captive Passeriformes was first described in the 1990s in the USA in Atlantic canaries [19] and zebra finches [20]. In 2000 and 2001, CV was also described in Atlantic canaries in Italy [19, 20]. In 2004, CV was found in a breeding aviary of Gouldian finches in the USA [23]. The molecular characteristics of zebra finch circoviruses were published by Rinder et al. in 2017 [24]. This virus showed 78% identity with the Gouldian finch CV [22]. In 2006, CV was detected in spleen samples from wild starlings *Sturnus vulgaris* and *Sturnus unicolor* found dead during an epidemic outbreak of septicemic salmonellosis in Northeastern Spain [25], as well as in Australian ravens (*Corvus coronoides*) [26]. In 2024, CV was described in Swinhoe’s white-eyes (*Zosterops simplex*), common hill mynas (*Gracula religiosa*) [27], and common raven (*Corvus corax*) [28].

Clinical signs of circovirus infection in Atlantic canaries include dullness, depression, lethargy, anorexia, abdominal swelling and reddening, distension of the gall bladder (‘black spot disease’), and failure to thrive [19, 21, 22, 29]. Morbidity and mortality rates range from 10% to 5% [22] to 90% and 100% [19], respectively. In most birds, the plumage is normal [10, 21, 29], but in the case described by Todd [22], feather disorders were observed. Histologically, lymphocyte necrosis and depletion in the bursa of Fabricius, necrosis of feather papillae, and oral epithelial cells are detected in some birds [19, 21, 29]. In zebra finches, the disease is characterized by feather loss, hepatocellular necrosis, and intracytoplasmic inclusion bodies in splenic lymphocytes and macrophages [20, 22].

In Gouldian finches, the mortality rate may reach 50%, including adult and young birds. Clinical signs include shortness of breath, anorexia, lethargy, and death within 2–5 days. Respiratory bacterial infections (*Escherichia coli* and *Klebsiella oxytoca*) have been reported. Problems with breeding were also observed, hatchability reduced by 20%, and high mortality, especially in birds younger than 6 months [23]. Histopathological findings include prominent depletion of lymphocytes in the bursa of Fabricius, and eosinophilic to basophilic spherical botryoid intracytoplasmic inclusion bodies in cells of monocyte/macrophage lineage. Additionally, eosinophilic intranuclear inclusion bodies are present in renal tubular epithelial cells. Mild to moderate lymphocyte depletion can also be observed in the thymus. Lymphocytic and plasmacytic inflammatory infiltrates are observed especially in the respiratory system, but also in the kidneys, adrenal glands, heart, pancreas, and intestinal lamina propria [23]. In wild birds, clinical signs have been described in Australian ravens with feather lesions similar to those that occur in psittacine beak and feather disease. Histopathological examination of abnormal feathers demonstrated, among others, dysplasia of developing follicles and a mild mixed inflammatory cellular infiltration in the pulp in some areas [26].

Vertical transmission of circoviruses has been confirmed in parrots and pigeons [30].

Diagnosis of CV infection is based, among others, on the detection of typical microscopic changes such as globular inclusion bodies in the cytoplasm of mononuclear cells of the follicles bursa of Fabricius, detection of circovirus-like particles in organ homogenates using negative contrast electron microscopy and by screening of circovirus-specific DNA using polymerase chain reaction (PCR) [24]. Studies of co-infection in captive Passeriformes with polyomaviruses and circoviruses have been performed, but with reference to co-infection with other infectious agents, such as *Mycobacterium genavense* [31] or canary bornaviuses [32]. Schmitz et al. [31] studied, among others, the co-occurrence of APyV and CV in 33 captive Passeriformes and found it only in one zebra finch (3%).

The aims of this study were to determine the prevalence of polyomaviruses and circoviruses in captive and wild Passeriformes in Poland, and to investigate the transmission of polyomaviruses and circoviruses through eggs in Atlantic canaries and Bengalese munias (*Lonchura striata domestica*).

## Materials and methods

### Postmortem examination and sample collection

Tissue samples were collected from 331 captive and wild Passeriformes of different ages, submitted to the Department of Pathology and Veterinary Diagnostics of Warsaw University of Life Sciences for postmortem examination by private individuals or institutions. Captive birds were sampled between 2006 and 2024, and wild birds were sampled between 2013 and 2024. From each bird, samples of liver, spleen, kidney, brain, and intestine were collected and stored between − 32 to -20 °C.

### Eggs and chicks sampling

One breeder of Atlantic canaries and another breeder of both Atlantic canaries and Bengalese munias, reported problems including embryo death during incubation and hatching, as well as chick mortality. Unhatched eggs, chicks, and juveniles or adults that had died from various causes were examined and sampled.

The affected Atlantic canaries samples were divided according to their age as follows: unfertilized eggs, embryos, chicks aged 1–5 days, 6–7 days, and 7–14 days, juveniles aged 1–8 months, and adults aged 1–2 years, 3–4 years, and 5–7 years. Bengalese munias were similarly grouped by age: unfertilized eggs, embryos, chicks, and adults. Eggs lacking visible embryonic structures or blood vessels were classified as ‘unfertilized’. Eggshells were washed with 3% hydrogen peroxide, cracked open, and a sample of yolk, albumen, and inner membrane was collected. Pooled samples of about 25 mg were prepared from eggs of a single clutch. Tissue samples were collected from embryos large enough for necropsy as described above, while for embryos in very early developmental stages, 25 mg of tissue was collected. Samples from eggs and chicks were placed in sterile plastic containers and frozen at -32 to -20 °C until DNA extraction.

### Histopathology

Samples from the liver, kidney, spleen, intestine, brain, and bursa of Fabricius (when present) were collected from thirty-five Atlantic canaries, one grey-crowned European goldfinch, two Bengalese munias and one common hill myna. These samples were fixed in 10% neutral buffered formalin, routinely processed for histopathological examination, and stained with haematoxylin and eosin (HE). The tissue sections were evaluated for the presence of intranuclear or intracytoplasmic viral inclusion bodies typical of polyomavirosis or circovirosis.

### DNA extraction, PCR and sequencing

Tissue samples collected during postmortem examination were pooled, containing fragments of each harvested organ (liver, spleen, kidney, brain, and the intestine from canaries), with a total weight of 25 mg. DNA was isolated from the pooled tissue samples, chicks, and embryos using the DNeasy Blood & Tissue Kits (QIAGEN, Germany) or Genomic Mini AX Tissue (A&A Biotechnology, Gdańsk, Poland), according to the manufacturer’s protocols. For eggs, DNA isolation was performed using the GeneMatrix Swab-Extract DNA Purification Kit (Eurx, Gdańsk, Poland), with approximately 25 mg of sample used in place of a swab; subsequent steps were performed according to the manufacturer’s instructions.

Nested PCR assays were used to identify viruses. Polyomaviruses were identified using the nested PCR assay developed by Johne et al. [12], while circoviruses were detected following the methodology developed by Halami et al. [33].

The products obtained from selected samples were then sequenced using a simple Sanger method. PCR products were purified with GeneMATRIX Agarose-Out DNA Purification Kit (EURx, Gdańsk, Poland) and sent for Sanger sequencing to Genomed (Warsaw, Poland).

Analysis of genetic sequences of selected circovirus and polyomavirus isolates was performed using FinchTV software v.1.4.0 (Geospiza Inc., 2004–2012) and The Molecular Evolutionary Genetics Analysis version 12 (MEGA 12) [34] software.

**Fig. 1:**
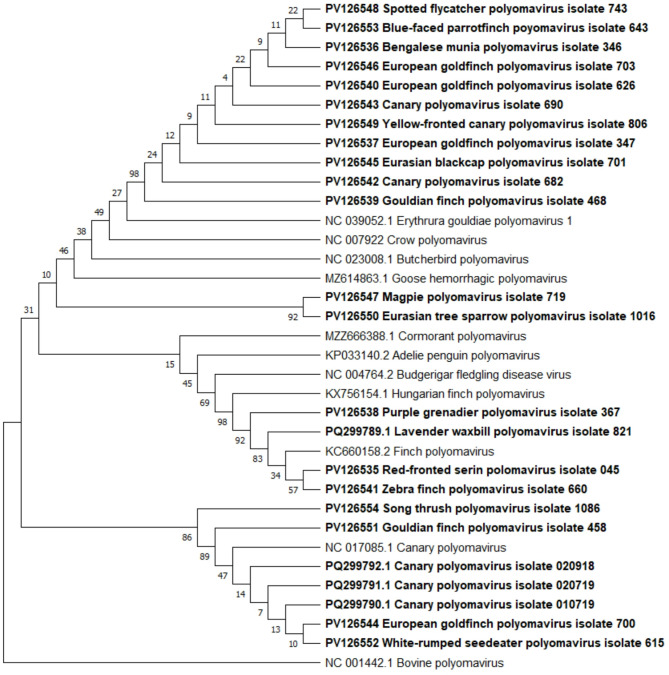
The relationships between the avian polyomaviruses strains detected in this study (in bold), Crow polyomavirus, Erythrura gouldiae polyomavirus 1, Goose hemorrhagic polyomavirus, Cormorant polyomavirus, Adelie penguin polyomavirus, Budgerigar fledgling disease virus, Hungarian finch polyomavirus, Finch polyomavirus, Canary polyomavirus and Bovine polyomavirus (root) – whose sequences were obtained from the GenBank database – are based on partial nucleotide sequences of the Major capsid protein (*VP1*) gene. Phylogenetic analysis was conducted using the Neighbor-joining tree-building method with Maximum Composite Likelihood genetic distance model. Bootstrap values (1000 replicates) are indicated at the corresponding branches. GenBank accession numbers are provided for all sequences shown

**Fig. 2:**
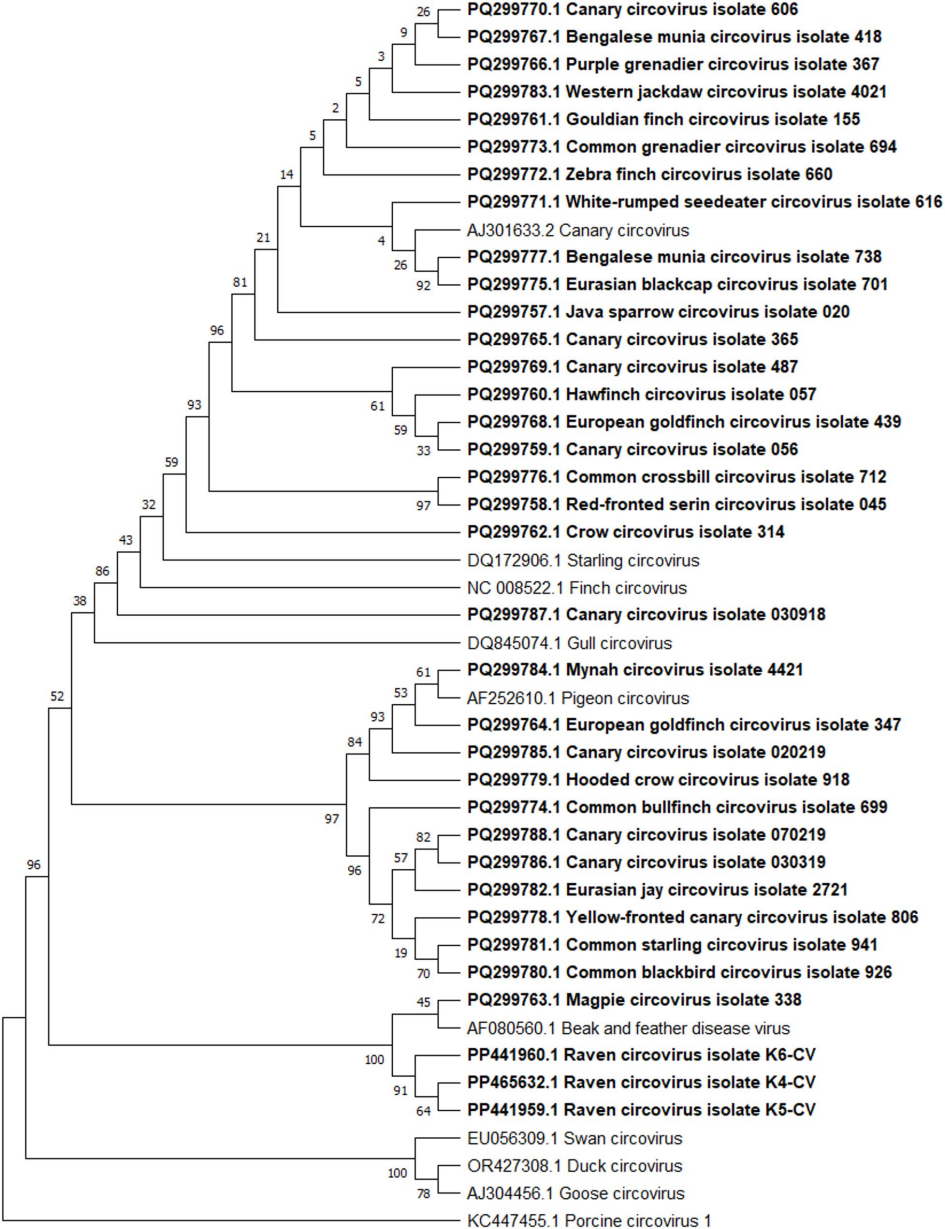
The relationships amongst circoviruses strains detected in this study (in bold), Canary circovirus, Starling circovirus, Finch circovirus, Gull circovirus, Pigeon circovirus, Beak and feather disease virus, Swan circovirus, Duck circovirus, Goose circovirus, Porcine circovirus 1 (root) – whose sequences were obtained from the GenBank database – are based on partial nucleotide sequences of the Replication-associated protein (*rep*) gene. Phylogenetic analysis was conducted using the Neighbor-joining tree-building method with Maximum Composite Likelihood genetic distance model. Bootstrap values (1000 replicates) are indicated at the corresponding branches. GenBank accession numbers are provided for all sequences shown

For phylogenetic analysis, the Neighbor-joining tree-building method with Maximum Composite Likelihood genetic distance model was applied following multiple sequence alignment by MUSCLE alignment of partial Major capsid protein *VP1* gene nucleotide sequences (polyomaviruses; Fig. 1) or partial Replication protein *(rep)* gene nucleotide sequences (circoviruses; Fig. 2). Sequences of Replication protein *(rep)* gene of Canary circovirus, Starling circovirus, Finch circovirus, Gull circovirus, Pigeon circovirus, Beak and feather disease virus, Swan circovirus, Duck circovirus, Goose circovirus, Porcine circovirus 1 and Major capsid protein *VP1* gene of Crow polyomavirus, Erythrura gouldiae polyomavirus 1, Goose hemorrhagic polyomavirus, Cormorant polyomavirus, Adelie penguin polyomavirus, Budgerigar fledgling disease virus, Hungarian finch polyomavirus, Finch polyomavirus, Canary polyomavirus and Bovine polyomavirus, derived from the GenBank database, were included in the analysis. Support for phylogenies was measured by bootstrapping 1000 replicates [35].

### GenBank accession

Sequences generated during this study have been submitted to GenBank. Accession numbers for circoviruses: PQ299757.1-PQ299788.1, PP465632.1, PP441960.1, PP441959.1. Accession numbers for polyomaviruses: PV126535.1-PV126554.1, PQ299789.1-PQ299792.1.

## Results

### Postmortem samples and PCR results

**Fig. 3:**
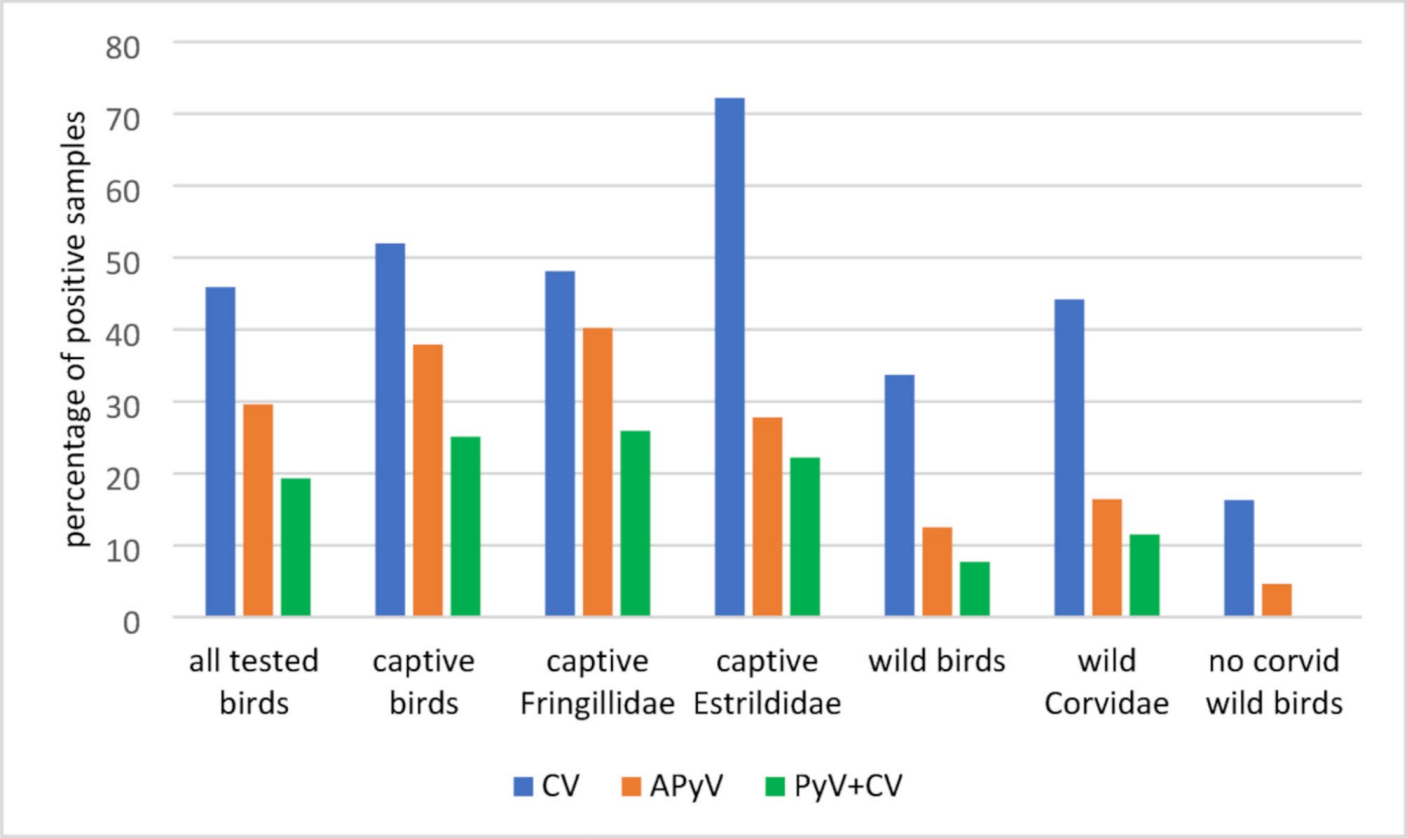
Percentage of positive results for avian polyomavirus (APyV), circovirus (CV), and co-infection with both viruses in captive and wild birds, with particular emphasis on captive Fringillidae and Estrildidae, and wild Corvidae

**Fig. 4:**
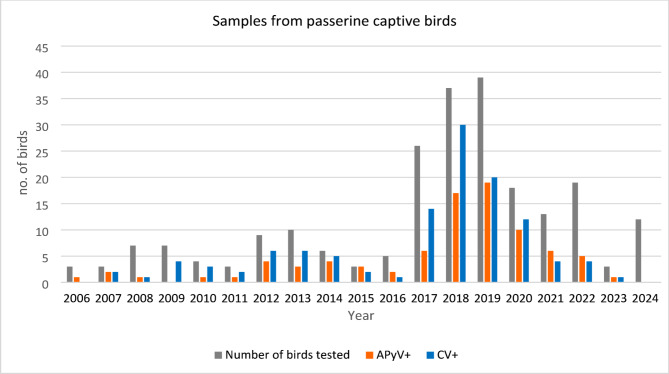
Number of captive Passeriformes tested between 2006 and 2024, and number of birds positive for circovirus (CV) and avian polyomavirus (APyV) DNA

**Fig. 5:**
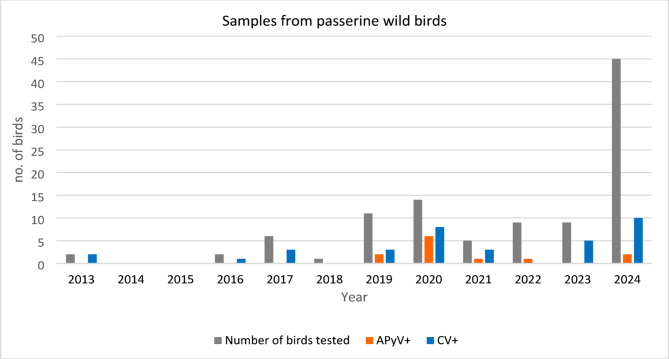
Number of wild Passeriformes tested between 2013 and 2024, and number of birds positive for circovirus (CV) and avian polyomavirus (APyV) DNA

From the 331 Passeriformes included in this study, 227 were captive birds and 104 were wild birds. The birds belonged to 45 species and 12 Families (Table 1). APyV was identified in 98 birds (29,6%) and CV was identified in 152 birds (45.9%). In addition, 64 birds were co-infected with APyV and CV (19,3%). A higher infection rate was found in the population of captive birds than in wild birds (Figs. 3, 4 and 5). In captive birds, the first APyV infection was detected in 2006 in a canary, while the first positive sample for circovirus was identified in 2007 in canaries and the common hill myna.

**Table 1:** Detections of avian polyomavirus (APyV) and circoviruses (CV) DNA in captive and wild passerine birds by species

Species	Scientific name	Family	Total birds tested	APyV-positive birds	CV-positive birds	Birds co-infected with APyV and CV	APyV and CV negative birds
Atlantic canary	*Serinus canaria*	Fringillidae	167	59	72	35	72
Red-fronted serin	*Serinus pusillus*	Fringillidae	4	1	4	1	0
White-rumped seedeater	*Serinus leucopygius*	Fringillidae	5	5	4	4	0
Yellow-fronted canary	*Serinus mozambicus*	Fringillidae	1	1	1	1	0
European goldfinch	*Carduelis carduelis*	Fringillidae	7	6	7	6	0
Atlantic canary x European goldfinch	*Serinus canaria/ Carduelis carduelis*	Fringillidae	4	3	2	1	0
European Goldfinch [Grey-crowned]	*Carduelis carduelis caniceps*	Fringillidae	1	1	1	1	0
Eurasian bullfinch	*Pyrrhula pyrrhula*	Fringillidae	3	0	3	0	0
Hawfinch	*Coccothraustes coccothraustes*	Fringillidae	1	0	1	0	0
Common redpoll	*Acanthis flammea*	Fringillidae	2	0	0	0	2
Red crossbill	*Loxia curvirostra*	Fringillidae	2	0	1	0	1
Eurasian chaffinch	*Fringilla coelebs*	Fringillidae	1	0	0	0	1
Bengalese munia	*Lonchura striata domestica*	Estrildidae	19	4	15	4	4
Java sparrow	*Lonchura oryzivora*	Estrildidae	3	0	1	0	2
Zebra Finch	*Taeniopygia guttata*	Estrildidae	4	1	4	1	0
Gouldian finch	*Erythrura gouldiae*	Estrildidae	3	2	3	2	0
Blue-capped cordon-bleu	*Uraeginthus cyanocephalus*	Estrildidae	1	1	1	1	0
Purple grenadier	*Uraeginthus ianthinogaster*	Estrildidae	1	0	1	0	0
Lavender waxbill	*Estrilda caerulescens*	Estrildidae	1	1	0	0	0
Violet-eared waxbill	*Uraeginthus granatinus*	Estrildidae	1	0	0	0	1
Blue-faced parrot-finch	*Erythrura trichroa*	Estrildidae	3	1	1	0	1
House sparrow	*Passer domesticus*	Passeridae	4	0	0	0	4
Eurasian tree sparrow	*Passer montanus*	Passeridae	2	1	0	0	1
Great tit	*Parus major*	Paridae	6	0	0	0	6
Marsh tit	*Poecile palustris*	Paridae	1	0	0	0	1
Coal tit	*Periparus ater*	Paridae	1	0	0	0	1
Eurasian wren	*Troglodytes troglodytes*	Troglodytidae	1	0	0	0	1
Eurasian Blackcap	*Sylvia atricapilla*	Sylviidae	1	0	0	0	1
Thrush nightingale	*Luscinia luscinia*	Muscicapidae	1	0	0	0	1
European robin	*Erithacus rubecula*	Muscicapidae	3	0	0	0	2
Common redstart	*Phoenicurus phoenicurus*	Muscicapidae	1	0	0	0	1
Spotted flycatcher	*Muscicapa striata*	Muscicapidae	1	0	0	0	1
Goldcrest	*Regulus regulus*	Regulidae	1	0	0	0	1
Eurasian golden oriole	*Oriolus oriolus*	Oriolidae	2	0	0	0	2
Common blackbird	*Turdus merula*	Turdidae	3	0	1	0	2
Song thrush	*Turdus philomelos*	Turdidae	2	1	0	0	1
Fieldfare	*Turdus pilaris*	Turdidae	1	0	0	0	1
Hill myna	*Gracula religiosa*	Sturnidae	2	0	1	0	1
European starling	*Sturnus vulgaris*	Sturnidae	3	0	1	0	2
Western jackdaw	*Corvus monedula*	Corvidae	11	3	4	3	7
Eurasian jay	*Garrulus glandarius*	Corvidae	2	2	1	1	0
Eurasian magpie	*Pica pica*	Corvidae	10	4	4	2	4
Hooded crow	*Corvus cornix*	Corvidae	29	0	9	0	20
Rook	*Corvus frugilegus*	Corvidae	3	1	3	1	0
Raven	*Corvus corax*	Corvidae	6	0	6	0	0
**Total**			**331**	**98**	**152**	**64**	**145**

The highest rate of circoviral infection was observed in Estrildidae (72.2%), followed by Fringillidae (48.1%) and Corvidae (44.2%). Polyomavirus infection occurred in the highest percentage of domestic Fringillidae (39.7%). The lowest rate of viral infection was found in wild Passeriformes not belonging to the Corvidae family. APyV and CV co-infection was not observed in no corvid wild passerine birds. The highest percentage of coinfections occurred in captive Fringillidae (Fig. 3).

### Clinical and necropsy findings vs. PCR

Most birds showed clinical signs (when clinical information was available) and post-mortem changes that were either nonspecific or typical of other diseases. Notable changes are described below.

In one of the breeding aviaries of canaries from which unhatched eggs and chicks were also collected for vertical transmission tests, in 2015 during polyomavirosis outbreak [10], a 5–20% decrease in hatchability was observed, young birds died at about 6 weeks of age. Prolonged molting and polyuria were observed in some young and adult birds. At that time, canaries in this aviary generally did not live longer than 2 years. Post-mortem examination revealed enlargement of the liver, kidneys, and spleen. In the second breeder of canaries and Bengalese munias from whom unhatched eggs, chicks, and young and adult birds were collected for vertical transmission tests, a decreased hatchability was observed, and molting disorders in two canaries coinfected with APyV and CV.

Co-infection with both viruses was found in three canaries with upper beak hypertrophy, in the fourth canary with this condition, only CV infection was found. Skin lesions in the form of hyperkeratosis and feather loss were noted in one magpie infected with circovirus. Lesions similar to lymphocytic leukemia were noted in two Bengalese munias and one zebra finch, but in these individuals, APyV infection was not detected, only a zebra finch and one Bengalese munia were infected with CV.

### Histopathology vs. PCR

**Fig. 6:**
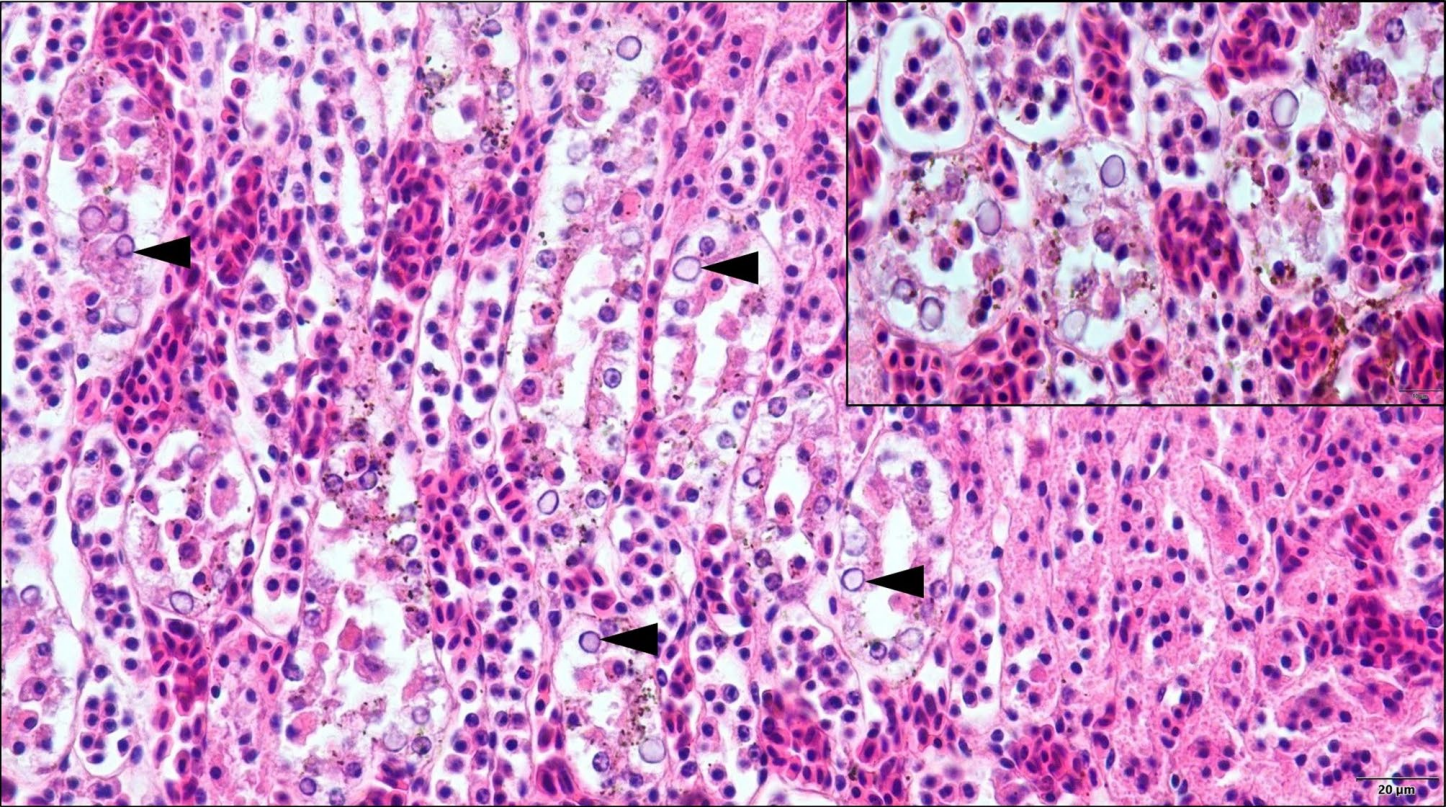
Photomicrograph of the kidney of an Atlantic canary (*Serinus canaria*) infected with avian polyomavirus (APyV), highlighting the presence of APyV intranuclear inclusions in renal tubular epithelial cells (arrowheads). The APyV inclusion bodies are round, large, homogenous, amphophilic to lightly basophilic with a glassy appearance, leading to karyomegaly, margination of nuclear chromatin, and are often associated with renal tubular epithelial necrosis (HE, 400x). Closer view of the affected tubules is shown on the insert (HE, 1000x oil immersion)

Histopathology was performed in tissue samples from 39 birds, being 35 Atlantic canaries, one grey-crowned European goldfinch, two Bengalese munias, and one common hill myna. From these, APyV intranuclear inclusion bodies in renal tubular epithelial cells were only observed in two Atlantic canaries (Fig. 6), which were from the same breeding facility, that died during an outbreak of the polyomavirosis [10]. In one of these canaries, both APyV and CV DNA were found, while in the other, only APyV DNA was detected. In the remaining 33 canaries without histopathological evidence of inclusion bodies, APyV DNA was found in 13 birds, CV DNA was found in three birds, and co-infection with APyV and CV was detected in five birds. The grey-crowned European goldfinch was co-infected with APyV and CV, but no inclusion bodies were observed (this bird died of atoxoplasmosis). In the remaining birds examined, no inclusion bodies typical of APyV or CV were detected.

### Vertical transmission of APyV and CV

Table 2 summarizes the results of the vertical transmission study of APyV and CV in Atlantic canaries and Bengalese munias. In Atlantic canaries, CV DNA was identified in 26 out of 33 unfertilized eggs and in 10 out of 17 embryos tested. In Bengalese munias, CV DNA was identified in 10 out of 16 unfertilized eggs and in all 5 chicks tested. These findings indicate that vertical transmission of CV occurs in Atlantic canaries and Bengalese munias. APyV DNA was not detected in any of the unhatched eggs from either species tested, nor in Bengalese munia chicks and Atlantic canary chicks younger than 7 days old.

**Table 2:** Detection of circovirus (CV) and avian polyomavirus (APyV) DNA in samples from Atlantic Canaries (*Serinus canaria*) and Bengalese munias (*Lonchura striata domestica*), grouped by age

Species	Age group	Total no. of samples tested	CV-positive samples	APyV-positive samples	Samples co-infected with APyV and CV
**Atlantic canaries**	Unfertilized eggs*	33	26 (79%)	0 (0%)	0
	**Embryos**	17	10 (59%)	0 (0%)	0
	**Chicks**,** 1–5 days**	5	2 (40%)	0 (0%)	0 (0%)
	**Chicks**,** 5–7 days**	8	0 (0%)	0 (0%)	0 (0%)
	**Chicks**,** 7–14** **days**	4	1 (25%)	2 (50%)	1 (25%)
	**Juveniles**,** 1–8 months**	19	7 (37%)	9 (47%)	1 (5%)
	**Adults**,** 1–2 years**	21	14 (67%)	11 (52%)	8 (38%)
	**Adults**,** 3–4 years**	12	9 (75%)	4 (33%)	4 (33%)
	**Adults**,** 5–7 years**	6	2 (33%)	3 (50%)	1 (17%)
**Total no. of Atlantic** **canaries**		**140**	**73**	**29**	**15**
**Bengalese munias**	Unfertilized eggs*	16	10 (62,5%)	0 (0%)	0 (0%)
	**Embryos**	5	5 (100%)	0 (0%)	0 (0%)
	**Chicks**	4	4 (100%)	0 (0%)	0 (0%)
	**Adults**	5	5 (100%)	3 (60%)	3 (60%)
**Total no. of Bengalese munias**		**29**	**23 (79**,**3%)**	**3 (10**,**3%)**	**3 (10**,**3%)**

*Unfertilized egg: eggs lacking visible embryonic structures or blood vessels

### Analyses of genetic sequences

The relationships amongst selected circovirus and polyomavirus strains detected in this investigation and submitted to GenBank are shown in Fig. 1 and in Fig. 2 as cladograms. Some of the Canary polyomavirus isolates divide long distance. Amongst polyomaviruses it has been shown for not only for our sequences (PV126542 and PQ299790-PQ299792) but cladogram shows also long distance between Canary polyomavirus sequence derived from GenBank database (NC_017085.1) and sequence obtained in described study (PV126543). Similar situation has been observed amongst circoviruses isolates – there is long distance between some isolates (especially seen for Canary circovirus – PQ299770.1, PQ299765.1, PQ299769.1, PQ299759.1, PQ299787.1, PQ299785.1, PQ299788.1, PQ299786.1) but also between some sequences obtained in this study and Canary circovirus sequence derived from GenBank database (AJ301633.2). About isolates obtained from other species it is worth to mention that polyomavirus isolates obtained from red-fronted serin (PV126535), zebra finch (PV126541) and lavender waxbill (PQ299789.1) were most closely related to Finch polyomavirus (KC660158.2) which sequence has been derived from GenBank database. Circovirus relationship analysis showed that isolates obtained from ravens (PP441960.1, PP441959.1, PP465632.1) and magpie (PQ299763.1) were most closely related to Beak and feather disease virus (AF080560.1) which sequence has been derived from GenBank database.

## Discussion

The studies carried out confirm the presence of circovirus and polyomavirus infections and co-infections in Poland in both captive and wild passerine birds. In wild birds, these viruses have been observed more often in corvids. Circovirus infections in corvids have already been described by other authors in Australian ravens, with plumage changes resembling viral beak and feather disease [26]. In the corvids we studied, feather abnormalities were found in only one magpie, and circovirus was found in both juvenile and adult birds. The occurrence of circoviruses and avian polyomaviruses, among others, has been studied in parrot and passerine bird aviaries in Germany [31]. This study in passerine birds showed circovirus infections in only one zebra finch breeding aviary, in which 3% of the birds were positive, out of 4 passerine bird aviaries tested. Polyomavirus infection was found in three out of four aviaries, and while Eurasian goldfinches were positive in 40% of the birds tested, zebra finches were infected in 96 -100% of the birds tested [31]. In our study of birds delivered from aviaries infected with both CV and APyV, the prevalence of CV infections in dead birds was higher than that of APyV. In the case described by Shuster et al. [15], polyomavirus infection in zebra finches was found to be responsible for lymphoma-like lesions. In the group of birds we studied, lymphoma-like tumours were observed in two Bengalese munia and one zebra finch. However, PCR testing for APyV was negative in all these birds.

APyV and CV co-infections were found in 1 zebra finch (3% of Passeriformes examined) examined post-mortem by Schmitz et al. [31] in the already mentioned Passeriformes. More studies on the phenomenon of co-occurrence of these viruses have been performed in parrots. In samples collected from live parrots co-infections ranged from 0.8% in captive psittacine birds in Chile [45] to 12.6% in duplex TaqMan probe-based real-time PCR in Korea [46]. However, it is difficult to compare the results from samples from live birds and additionally from other orders to those obtained from dead birds, as was the case in our study. The clinical significance of co-infections in parrots has been described in the context of the occurrence of polyomavirosis symptoms in parrots resistant to the disease due to their age or species, and in which the disease occurred due to simultaneous infection with parrot circovirus [17]. In the birds we studied from breeding aviaries where clinical polyomavirosis occurred, co-infection was not observed in all dead birds. Most canaries in which feather and beak growth abnormalities were observed were co-infected with APyV and CV.

Our studies, similarly to those of other authors [2], have shown low sensitivity of histopathological examination compared to molecular tests. However, it should be remembered that typical microscopic changes confirm the actual pathogenicity of the detected virus.

Sequence analyses based on CV partial replication protein (rep) gene nucleotide sequences have shown a considerable amount of mixing of circoviruses between species, which may indicate that some circoviruses may not be species-specific but exhibit natural spillover infection, as shown by the study conducted by Nath et al. [36]. However, because our sequences do not constitute complete genomes in both CV and APyV, it is important to have in mind that phylogenetic analysis could be inadequate, and further investigations of obtained isolates are warranted.

Transmission of CV and APyV via eggs has not been previously studied in Passeriformes. Our study demonstrated vertical transmission of CV, whereas APyV genetic material was not present in any of the eggs or chicks examined. Vertical transmission of circoviruses has been confirmed in parrots, by Rahaus et al., where viral DNA was found in 35.3% of non-embryonated and 20% of embryonated eggs [30], and ducks [37]. More questionable is the transmission of avian polyomaviruses. In ducks, vertical transmission of goose hemorrhagic polyomavirus has been shown to be very low, with qPCR detecting genetic material of the virus in 2 out of 25 eggs, while conventional PCR did not detect its presence. Book sources citing data in conference reports show early embryonic mortality and sudden death in 2- to 3-day-old nestling finches, as well as in fledgling and young adult finches [30, 38–41]. Our study showed the presence of the virus in canaries as young as 7 days of age, i.e., those that may have become horizontally infected. However, the PCR test for APyV was negative in these birds.

Similar observations were made by Phalen, who observed no inclusion bodies in parrot chicks younger than one week [17]. Phalen also suggests that, despite existing reports of the possibility of parrot polyomavirus transmission through the egg [42, 43], there is only very limited and circumstantial evidence that vertical transmission occurs [17]. In Bengalese munia, there was also no evidence of APyV in infertile eggs, embryos, chicks, or juveniles up to 30 days of age. However, the number of hatchlings examined was relatively low, which is a major limitation of this study.

## Conclusions

Avian circovirus and polyomavirus infections have been diagnosed in an increasing number of passerine species, both captive and wild, over a 19-year study period in Poland. Although clinical disease has been described in captive birds, little is still known about the clinical manifestations of these infections in wild birds or the potential threat they pose to avifauna. The incidence of APyV and CV co-infections in the Passeriformes studied was higher than previously reported in other bird species. Vertical transmission of CV in Atlantic canaries and Bengalese munias has been confirmed, whereas vertical transmission of avian polyomaviruses has not been detected.

Due to the widespread of APyV and CV among passerine birds, future research should focus on investigating possible cross-species infections, with particular attention paid to cross-infection between wild and captive populations. Efforts to eliminate these infections from breeding aviaries of these birds should also be focused on, among others, vaccinations [44, 45] and biosecurity diagnostic methods available to breeders to prevent the introduction of infected individuals to breeding.

## Data Availability

The data of this study are included in the manuscript. The data is available up on request from the corresponding author. Accession numbers of APyV and CV strains submitted to GenBank are located on the phylogenetic trees (Figs. 1 and 2).

## Abbreviations

ApyV: Avian polyomavirus
FPyV: Finch polyomavirus
CV: Circovirus
MUSCLE: Multiple Sequence Comparison by Log-Expectation
VP1: Viral protein 1
Rep: Replication protein
